# Bone Marrow-Derived Endothelial Progenitors Expressing Delta-Like 4 (Dll4) Regulate Tumor Angiogenesis

**DOI:** 10.1371/journal.pone.0018323

**Published:** 2011-04-04

**Authors:** Carla Real, Leonor Remédio, Francisco Caiado, Cátia Igreja, Cristina Borges, Alexandre Trindade, Perpétua Pinto-do-Ó, Hideo Yagita, Antonio Duarte, Sérgio Dias

**Affiliations:** 1 Angiogenesis Laboratory, CIPM/Portuguese Institute of Oncology, Lisbon, Portugal; 2 Instituto Gulbenkian de Ciência, Oeiras, Portugal; 3 Centro de Química e Bioquímica, Faculdade de Ciências da Universidade da Universidade de Lisboa, Lisbon, Portugal; 4 Department of Immunology, Juntendo University School of Medicine, Tokyo, Japan; 5 CEDOC, Faculdade de Ciencias Médicas, Universidade Nova de Lisboa, Lisbon, Portugal; 6 CIISA, Faculdade de Medicina Veterinária, Technical University of Lisbon, Lisbon, Portugal; 7 Divisão de Biomateriais, INEB–Instituto de Engenharia Biomédica, Universidade do Porto, Porto, Portugal; National Cancer Institute, United States of America

## Abstract

Neo-blood vessel growth (angiogenesis), which may involve the activation of pre-existing endothelial cells (EC) and/or the recruitment of bone marrow-derived vascular precursor cells (BM-VPC), is essential for tumor growth. Molecularly, besides the well established roles for Vascular endothelial growth factor (VEGF), recent findings show the Notch signalling pathway, in particular the ligand Delta-like 4 (Dll4), is also essential for adequate tumor angiogenesis; Dll4 inhibition results in impaired, non-functional, angiogenesis and reduced tumor growth. However, the role of BM-VPC in the setting of Notch pathway modulation was not addressed and is the subject of the present report. Here we show that SDF-1 and VEGF, which are produced by tumors, increase Dll4 expression on recruited BM-VPC. Mechanistically, BM-VPC activated, in a Dll4-dependent manner, a transcriptional program on mature EC suggestive of EC activation and stabilization. BM-VPC induced ICAM-2 and Fibronectin expression on EC, an effect that was blocked by a Dll4-specific neutralizing antibody. *In vivo*, transplantation of BM-VPC with decreased Dll4 into tumor-bearing mice resulted in the formation of microvessels with decreased pericyte coverage and reduced fibronectin expression. Consequently, transplantation of BM-VPC with decreased Dll4 resulted in impaired tumor angiogenesis, increased tumor hypoxia and apoptosis, and decreased tumor growth. Taken together, our data suggests that Dll4 expression by BM-VPC affects their communication with tumor vessel endothelial cells, thereby modulating tumor angiogenesis by affecting vascular stability.

## Introduction

Besides sprouting of pre-existing endothelium, tumor angiogenesis may require also the contribution of bone marrow-derived endothelial progenitor cells (EPC) [1], previously shown to be recruited into tumors (reviewed in [2]) and proven essential for tumor angiogenesis [3]. However, the mechanisms by which EPC contribute towards angiogenesis still remain largely undisclosed, mainly because of the very low number of EPC found within tumor biopsies, in or around vessels [4], [5]. Moreover, the identity of true “EPC” with proangiogenic functions is still under intense scrutiny (Reviewed in [6]). For instance, recent reports have elegantly shown that bone marrow-derived myelomonocytic cells but not “EPC” have proangiogenic potential during spontaneous tumor formation and growth [7]. Therefore, bone marrow-derived cells other than pure “EPC” may also play a crucial role during tumor angiogenesis and growth; a more detailed molecular and functional definition of EPC and of other bone marrow-derived cells with proangiogenic potential is clearly needed.

Notch signalling is crucial during embryonic development, for the differentiation of different tissues, and also in adult homeostasis. Notch ligands (Delta like 4, Dll4) and receptors (Notch 1 and Notch 4) have been shown to be involved in the differentiation and function of the vasculature, during embryogenesis and in adults. In detail, Dll4 deficient mice have severe vascular defects, similar to Notch 1 and 4 knock-outs [8], [9]. More strikingly, in inbred genetic backgrounds Dll4 heterozygous embryos (in haploinsufficiency) die at mid gestation due to severe vascular effects, highlighting its importance in vasculogenesis and its role over other members of the Notch pathway [10], [11]. Notably, similar vascular defects are observed in haploinsufficient VEGF mouse embryos [12]. Consequently, a putative role for Dll4 in tumor angiogenesis has been under intense scrutiny [13], [14], [15], [16]. However, it is still unclear whether Dll4 is only expressed on tumor vessels or other cell types, and what role it plays during angiogenesis.

Given its crucial role in modulating vessel formation and function, in the present study, we hypothesized Dll4 expressed on bone marrow derived vascular progenitor cells (BM-VPC) might play a role in tumor angiogenesis, either by activating the pre-existing endothelium or by promoting vessel stabilization following sprouting and proliferation.

## Results

### SDF1 and VEGF induce Dll4 expression on BM-VPC

BM-VPC have been shown to be recruited into the peripheral blood in response to VEGF and SDF1 produced by tumors and to express Notch signalling pathway components, such as Dll4 [17]. We investigated whether these factors were able to regulate the expression of Dll4 on BM-VPC.

**Figure 1:**
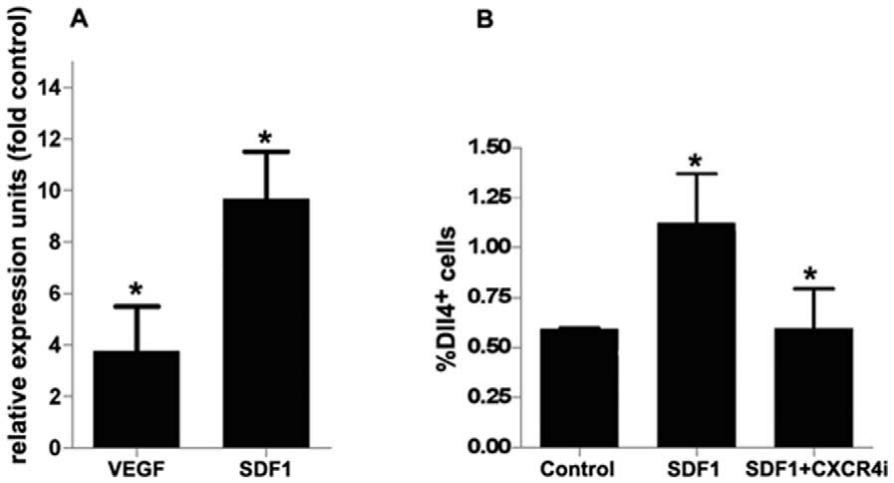
Dll4 expression on BM-VPC is induced by VEGF and SDF1. A. Expression of Dll4 in BM-VPC was detected by RT-PCR. B. BM-VPC were incubated with SDF1 (50 ng/ml) or SDF1 and CXCR4 inhibitor (5 ug/ml) The number of Dll4 positive cells was quantified by flow cytometry using anti-Dll4 mouse specific. Each experiment was performed in triplicate and the mean represented n = 3. (values show the mean plus s.e.m. *: P<0.05).

BM-VPC were cultured in presence of VEGF or SDF1 for 18 hours. Dll4 expression was analysed by RQ-PCR and flowcytometry. Dll4 expression was significantly increased on BM-VPC cultured in presence of both VEGF and SDF1 compared to the control condition (Figure 1A), although comparatively SDF1 induced a greater increase in Dll4 expression. We also verified that Dll4 regulation by SDF1 was mediated via its receptor, CXCR4. We incubated BM-VPC with SDF1 alone or in the presence of a CXCR4 inhibitor for 18 h and analysed the cells by flowcytometry. As shown in Figure 1B, CXCR4i prevents DLL4 induction by SDF1 (Figure 1B). The results show that the expression of Dll4 on BM-VPC is induced by VEGF and by SDF1; the latter effect is mediated through its receptor, CXCR4.

### Dll4 induces a vascular activation and stabilization program on mature EC *in vitro*

**Figure 2:**
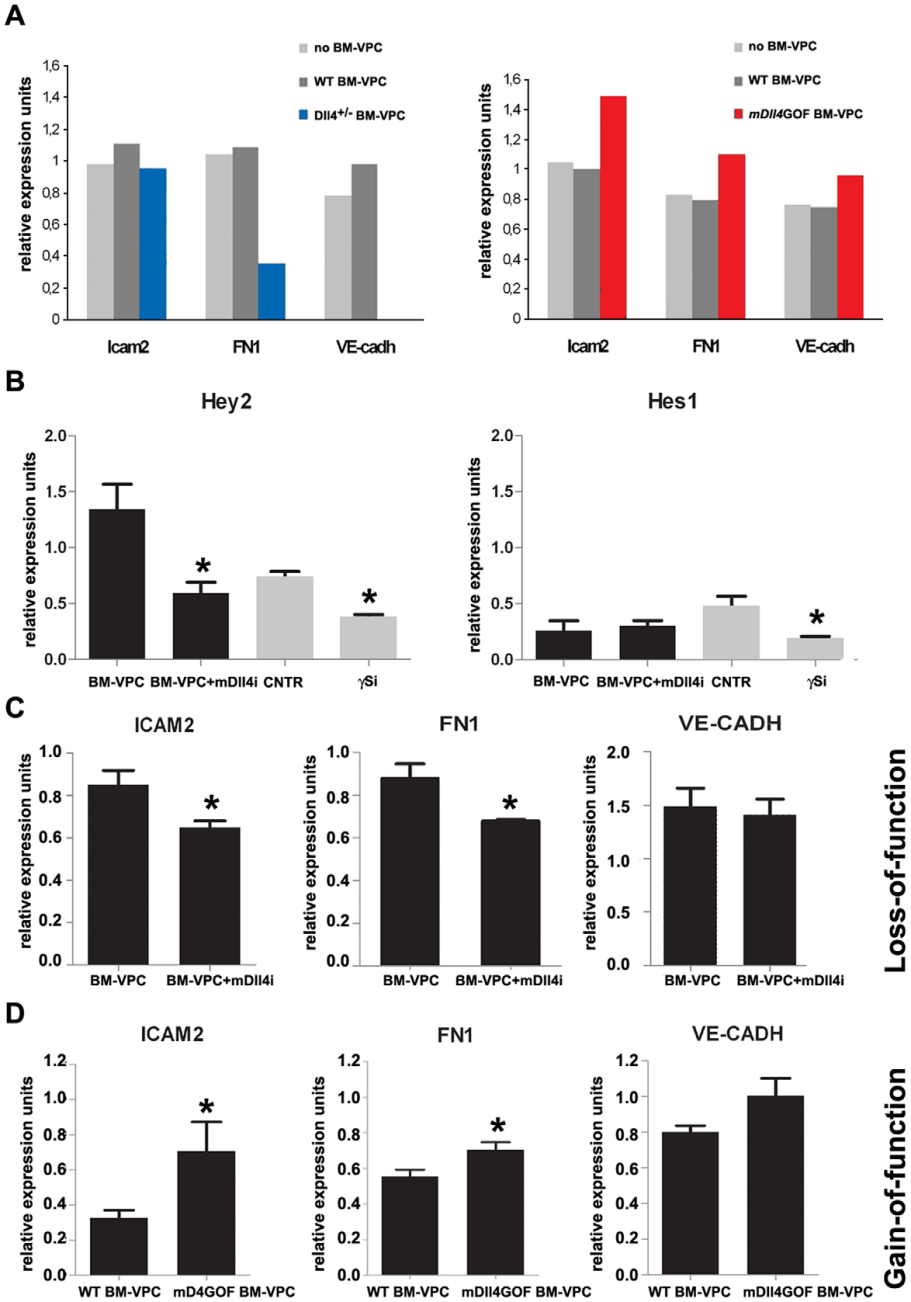
Dll4 expressed on BM-VPC stimulate a vascular activation and stabilization program on endothelial cells. A. Genes differently expressed on HUVEC after co-cultered with BM-VPC-WT, BM-VPC-Dll4 or mDll4GOF BM-VPC. These results were obtained from one hybridization using pooled samples from 3 experiments. B. Expression of Hey2 and Hes1 in HUVEC co-cultured with mouse isolated BM-VPC in presence of mouse specific neutralizing antibody for Dll4 (mDll4i) or γ-secretase inhibitor (γSi). C. ICAM2, FN1 and VE-Cadherin expression in HUVEC co-cultured with mouse BM-VPC or BM-VPC in presence of mouse specific neutralizing antibody for Dll4 (mDll4i). D. ICAM2, FN1 and VE-Cadherin expression in HUVEC co-cultured with mouse BM-VPC-WT or MDll4GOF BM-VPC. Gene expression was quantified by RT-PCR in B,C and D. (values show the mean plus s.e.m. *: P<0.05).

We hypothesized that DLL4, driven by BM-VPC, could signal on EC, inducing molecular changes that may lead to an angiogenic response in vitro and in vivo. To test this hypothesis, we cultured HUVEC together with BM-VPC isolated from mDll4 overexpression (mDll4GOF BM-VPC) or heterozygous (low expression) Dll4+/− (Dll4+/-BM-VPC) mice and used HUVEC co-cultured with WT BM-VPC as control. After o.n. contact, we removed BM-VPC and compared HUVEC gene expression using a microarray containing 113 genes related to EC biology. In particular, the expression of ICAM2, FN1 and VE-Cadherin increased in HUVEC cultured in presence of mDll4GOF BM-VPC and decreased in case of Dll4+/− BM-VPC (Figure 2A).

To further demonstrate that the regulation of these genes on EC was Dll4-specific we co-cultured HUVEC and mouse BM-VPC, in presence or absence of a mouse Dll4-specific inhibitor antibody (mDll4i). Hey2 and Hes1 quantification was used to confirm the activation state of the Notch signaling pathway on HUVEC. As shown in Fig. 2B, Dll4 inhibition on BM-VPC decreased Hey2 but not Hes1 expression by co-cultured HUVEC; in contrast, the gamma-secretase and Notch signaling inhibitor, GSI, inhibited both downstream Notch target genes (Figure 2B). Quantitative Q-PCR was used to determine the differences in the expression of ICAM2, FN1 and VE-Cadherin, in co-cultured HUVEC. Notably, FN1 and ICAM2 expression on induced by BM-VPC on HUVEC decreased in the presence of the Dll4 inhibitor (n = 3, P<0,05)(Fig. 2c). In contrast, VE-Cadherin expression was not altered by Dll4 inhibition (Figure 2C). Inversely, when HUVEC were co-cultured with mDll4GOF BM-VPC, the expression of FN1 and ICAM2 decreased, while VE-Cadherin was not altered (Figure 2D). Taken together, these in vitro data indicate that Notch signaling pathway activation of EC by Dll4 driven by BM-VPC induces gene expression changes on EC, namely of genes linked to endothelial activation and stability such as fibronectin and ICAM2.

### Dll4 expression by BM-VPC is essential for tumor angiogenesis and growth

**Figure 3:**
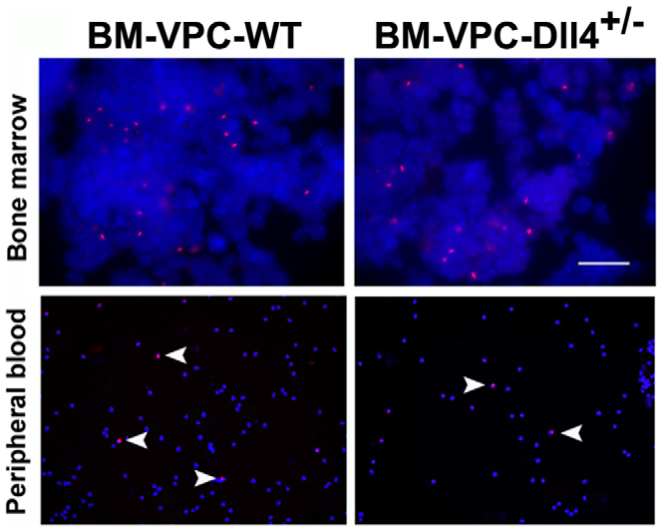
BM-VPC-Dll4 engraft bone-marrow of NOD-SCID mice and are recruited to peripheral blood during tumour growth. BM-VPC-WT and BM-VPC-Dll4 are equally present in bone-marrow and peripheral blood 15 days after inoculation of cells. BM-VPC were identified using FISH to Y chromosome.

Next, to test whether the role of Dll4-expressing BM-VPC was essential to tumor angiogenesis, we developed an in vivo approach in which we reconstituted NOD-SCID mice BM with BM-VPC obtained from Dll4+/− (heterozygous, with reduced Dll4 levels) or from WT (normal Dll4 levels) mice. As shown by fluorescent in situ hybridization using a Y chromosome probe, WT BM-PC and Dll4+/− BM-VPC engraft the bone marrow of irradiated recipient mice, are recruited into the peripheral blood 2 weeks after tumor implant and incorporate the tumor mass at approximately the same rates and frequency (Figure 3)

**Figure 4:**
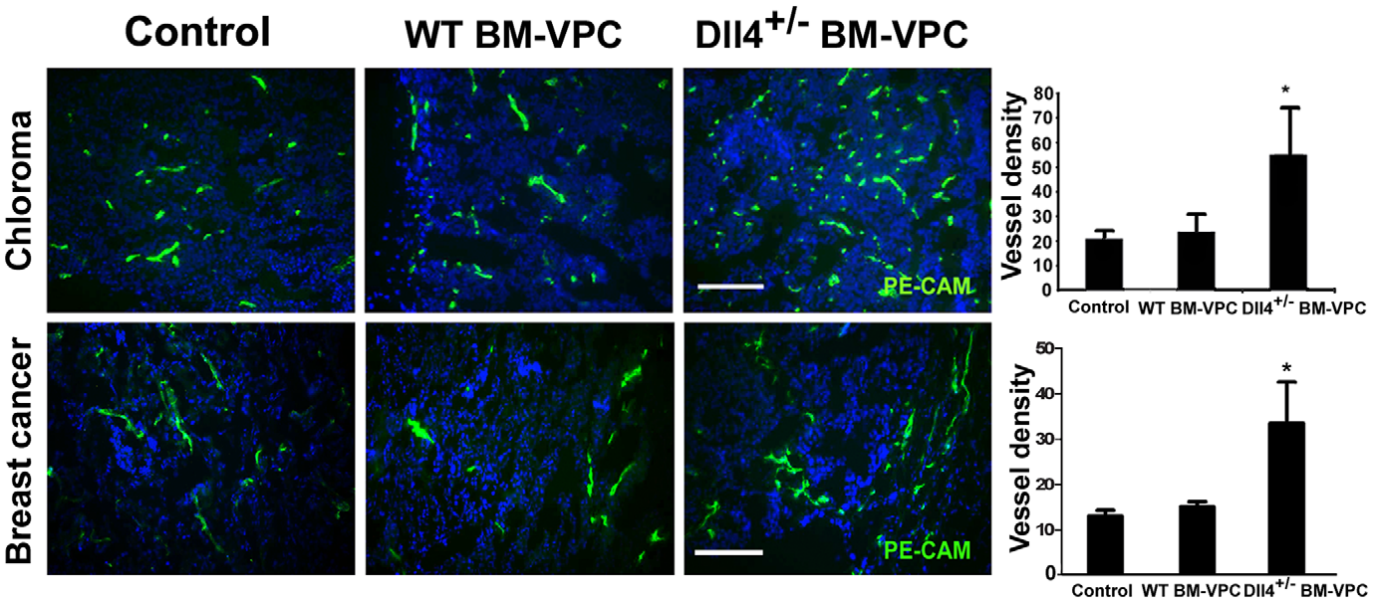
Dll4+/-BM-VPC increase tumour vessel density in tumors. Endothelial cells were identified by immunofluorescence using anti-CD31 (PE-CAM) in tumor cryosections from mice inoculated with BM-VPC-WT or BM-VPC-Dll4. Scale Bar indicates 250 µm. Graphs show the number of microvessels per section (values show the mean plus s.e.m. *: P<0.05).

Normal BM-VPC (male lin-flk1+) or from Dll4+/− mice (Dll4+/− BM-VPC) were used in transplantation experiments into recipient female mice. Next, we analysed tumor angiogenesis using a well established in vivo model of human chloroma (solid leukemia-derived tumor, previously shown to be very efficient at recruiting BM-VPC, [2]). Tumor microvessel density was determined by immunofluorescence staining against PE-CAM. As seen in Figure 4, Human tumors growing in mice transplanted with Dll4+/− BM-VPC had a significantly higher microvessel density than those with BM-VPC-WT.

**Figure 5:**
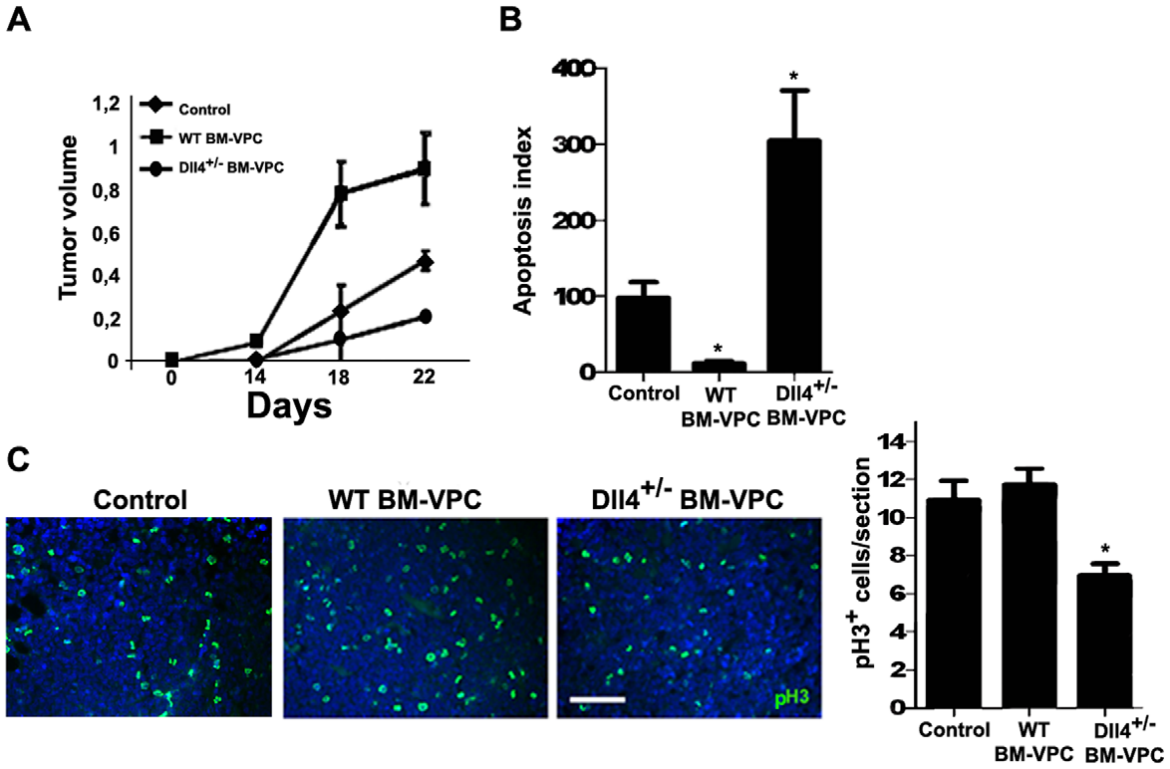
Dll4+/−BM-VPC tumors have a smaller growth rate than WT BM-VPC tumors, and increased apoptosis. A. Tumor volume ((LxW2)/2) in control, WT BM-VPC and Dll4+/− BM-VPC-Dll4 tumors. B. As determined by TUNEL staining, tumor apoptosis index 15 days after inoculation, in control, WT BM-VPC and Dll4+/− BM-VPC tumors. C. Tumor proliferation index (number of phosphor-histone 3 (pH3) per section) pH 3 staining in tumor cryosections. These quantifications were done in triplicate, in tumor sections obtained from 2 independent experiments. Scale bar indicates 150 µm. (values show the mean plus s.e.m. *: P<0.05).

However, if tumors were allowed to grow beyond 15 days, there was a significant growth delay of tumors in BM-VPC-Dll4+/− transplanted versus control mice or those with WT BM-VPC (Figure 5A). As determined by Tunel (to identify apoptotic cells) and phospho-histone H3 (which identifies proliferating cells) staining, tumors with Dll4+/− BM-VPC showed significantly a higher apoptosis index and lower proliferation rate than control and WT BM-VPC (Figure 5B–C).

### Dll4 expression by BM-VPC affects the stability of tumor vessels

**Figure 6:**
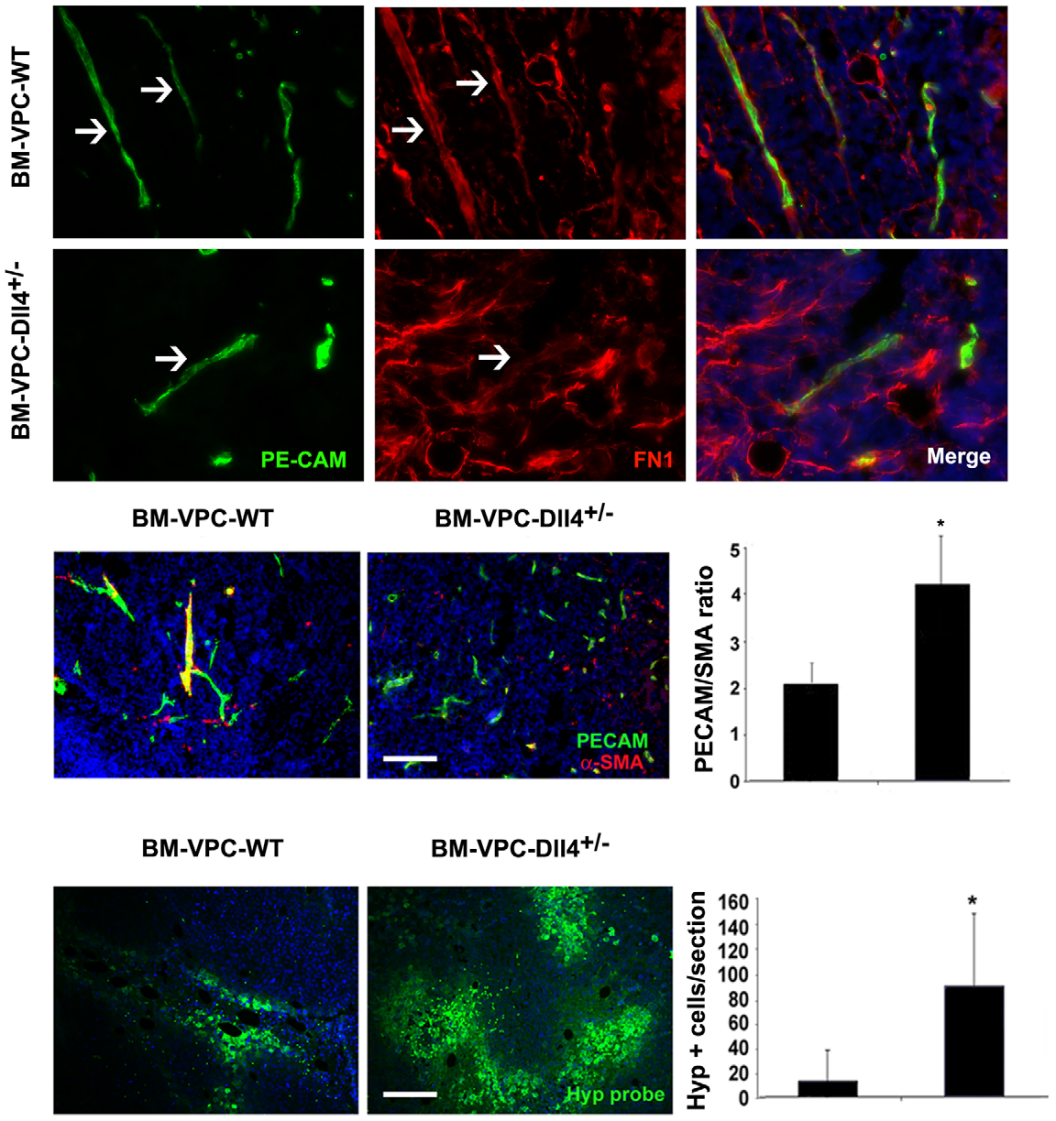
Dll4+/-BM-VPC regulates vessel stability in tumors. A. FN1 expression was detected by immunofluorescence (FN1) endothelial cells were stained using anti-PE-CAM antibody. B. Quantification of the number of PE-CAM+SMA+ and PE-CAM+SMA- vessels, (PECAM/SMA ratio of 2.2±0.9 versus 4.6±2.3, respectively, * P<0.05). Scale Bar represents 350 µm. C. Hypoxia index 15 days after inoculation (number of hipoxyprobe positive cells per section) (* P<0.05). Scale bars represent 350 µm.These quantifications were done in triplicate, in tumors from 2 independent experiments.

We assessed vessel stability as determined by immunofluorescence staining against fibronectin (FN1) and SMA (smooth muscle actin, which identifies pericytes). We observed that tumors with Dll4+/− BM-VPC had significantly lower FN1 content (Figure 6A) in the vessels and these had significantly less pericyte coverage, suggesting these would likely be more unstable (the PECAM/SMA ratio of the tumors is shown in Figure 6B; the higher the ratio the less pericyte/SMA coverage of tumor vessels). Moreover, as determined by staining tumor frozen sections with hypoxiprobe (which labels hypoxic tumor areas), tumors with Dll4+/− BM-VPC had significantly greater proportion of hypoxic areas, which strongly suggests that the vessels in these tumors were less functional (Figure 6C). Taken together, these data show the tumor vessels grown in BM Dll4+/− BM-VPC transplanted versus control mice or those with BM-VPC-WT are non-functional, leaky vessels.

### Dll4 expression on BM-VPC affects vessel stability in late stages of tumor growth

**Figure 7:**
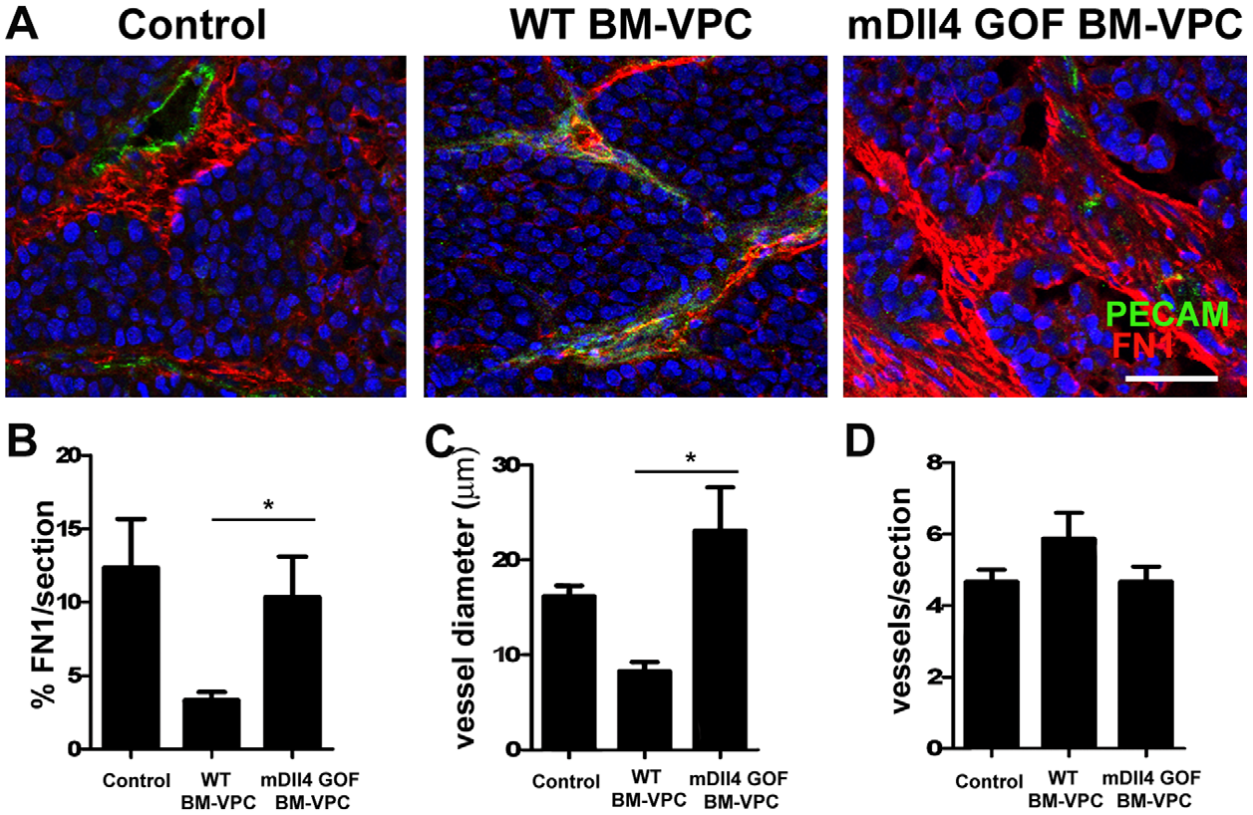
Increased expression of Dll4 in circulating blood cells regulates vessel stability in early stages of tumor development. A. Fibronectin 1 (FN1) and PECAM expression were detected by immunofluorescence. Scale bar indicates 50 µm B. FN1 staining quantification was performed using ImageJ. C. Vessel diameter was quantified measuring the smallest distance between two endothelial nuclei in opposing sides at more than 4 different vessel levels. D. Vessel density was obtained by counting the number of vessels per section. (values show the mean plus s.e.m. *: P<0.05).

Having shown the relevance of Dll4 expression on BM-VPC for EC behavior in vitro, next we investigated whether these effects were also observed in vivo during tumor growth and specifically dependent on Dll4 expression by BM-VPC using a xenograft tumor model of mouse breast carcinoma (HTH-k). For that we increased the number of circulating BM-VPC by administering mouse BM-VPC derived from Dll4GOF mice with induced Dll4 over-expression (mDll4GOF BM-VPC) or normal Dll4 expression (WT BM-VPC) after tumor establishment during a restricted period of time. We analysed subcutaneous tumor xenografts of mice injected at day 6, 8 and 10 with BM-VPC, compared with mice in the same experimental conditions but without BM-VPC administration (Figure 7).

To assess vessel stability we quantified Fibronectin 1 content in blood vessels of controls, WT BM-VPC and mDll4GOF BM-VPC tumors. We identified endothelial cells by immunostaining with PE-CAM. To assess vessel stability we quantified Fibronectin 1 content and blood vessel diameter in blood vessels of tumors. Fibronectin 1 expression and vessel diameter were significantly higher in tumors treated with mDll4GOF BM-VPC than WT BM-VPC (Figure 7A–7C). However, the vessel density and tumor volume (data not shown) were similar between controls, WT BM-VPC and mDll4GOF BM-VPC tumors (Figure 7D). Taken together, this data suggest that specific expression of Dll4 on BM-VPC modulates vessel morphology at tumor site, increasing fibronectin1 content.

## Discussion

Therapeutic approaches to target tumor angiogenesis have shown promising pre-clinical results [18], [19], [20], although some studies have reported significant side effects, while others have suggested better and more detailed molecular studies are still needed given the relatively modest therapeutic benefits observed [21], [22]. It is now well established that VEGF is not the only factor essential for tumor angiogenesis, and that other signalling pathways play a crucial role during the initial (sprouting) and the late (stabilization) phases of this intricate and complex process. Moreover, the role for, and the importance of, the endogenous (pre-existing) and the “external” (BM-recruited progenitors, termed VPC throughout this manuscript) factors that regulate endothelial cells during tumor angiogenesis is still largely undisclosed and has been the theme of some controversy.

Recent studies revealed that the Notch signalling pathway, and specifically its ligand Dll4, is crucial for adequate tumor angiogenesis [15], [23], [24]. In detail it was shown that therapeutic strategies aimed at neutralizing Dll4 binding to its receptor (with Dll4 neutralizing antibodies) resulted in inefficient angiogenesis due to vessel instability. A putative role for BM-VPC in the setting of Notch:Dll4 signaling during tumor angiogenesis has not been studied and was the subject of the present report.

Our results demonstrate that BM-VPC are activated during tumor growth by SDF1 and VEGF resulting in increased Dll4 expression. VEGF and SDF1 produced in the tumor microenvironment, and released systemically, have been implicated in BM-VPC recruitment and retention in perivascular sites [25]. In this paper, we demonstrate that these cytokines are also responsible for the activation of BM-VPC. It has already been described that SDF1 increases BM-VPC-dependent vasculogenesis [26]. However, the molecular mechanism responsible for this effect was unknown. Here we show that SDF1 is able to regulated Dll4 expression in BM-VPC altering the angiogenic response. Therefore, the Dll4 expression levels might be considered as a general marker for vascular responses, such as during tumor angiogenesis or vascular remodelling events.

Dll4 expression on EC activates the Notch signalling pathway resulting in the regulation of tumor angiogenesis in a VEGF-independent manner [23], [24], [27]. In the present paper, we show that BM-VPC also regulates Notch signalling activity on EC via Dll4 expression. Dll4 expressed by BM-VPC was able to regulate FN1 and ICAM2 (among other genes) expression on mature EC, suggesting it might modulate vessel stability and activation programs. As already described in the context of the Dll4 overexpression mutant, Notch signalling activation by Dll4 on EC was able to regulate the expression of several components of the extracellular matrix including FN1 [28], suggesting it modulates vessel stability by controlling the expression of ECM components of the basement membrane. Moreover, it was already shown that the overexpression of Dll4 on EC increases the expression of VE-cadherin, a cell-to-cell adhesion molecule [28]. Our results do not show any difference in endothelial cell VE-cadherin expression with our without Dll4 inhibition on BM-VPC. This might indicate that VE-Cadherin regulation on endothelial cells is a cell-autonomous mechanism, since in our experiments Notch activation by Dll4 expressed on EC was not tested. Besides promoting leukocyte adhesion during inflammatory responses, ICAM2 is also implicated in angiogenesis, where it was shown to promote tube formation [29]. Taken together, Dll4 expression on BM-VPC is able to activate mature EC, which strongly suggests the cross-talk between these 2 cell types is crucial for adequate tumor angiogenesis.

In vivo, we observed that reduction of Dll4 expression on BM-VPC, and subsequent transplantation into tumor bearing mice, decreased tumor vessel stabilization resulting in the formation of unstable vessels. Dll4 reduction on BM-VPC (Dll4+/− BM-VPC) and their transplantation into tumor-bearing mice resulted in the formation of unstable vessels, as evidenced by the reduced pericyte coverage, reduced Fibronectin expression; this led to appearance of more hypoxic areas and consequently decreased tumor growth. Vessel instability has been previously observed in other studies looking at the importance of Dll4 during tumor angiogenesis and also embryonic vasculogenesis [23], [24], [27], but this effect was not related to the contribution/involvement of BM-VPC. In addition, we also demonstrate that specifically modulating Dll4 expression on BM-VPC is sufficient to affect vascular stability of tumor vessels during a restricted period throughout tumor development. Globally, the results presented here show that gene expression alterations in the ligand Dll4 on BM-VPC regulate angiogenesis at the tumor site. Nevertheless, we cannot exclude the possible contribution/involvement of other Notch ligands in the communication between BM-VPC and resident tumor endothelial cells.

Taken together, our data further supports the concept that Dll4 expression and Notch signalling pathway activation must be tightly controlled to produce a normal and functional vessel network. Our data shows that BM-VPC may be essential in the control of this signalling pathway at different stages of tumor angiogenesis. Dll4 expression by BM-VPC may therefore be considered as an important regulator of vessel stabilization, essential for tumor angiogenesis but also in other vascular pathologies.

## Materials and Methods

### Mouse strains

Dll4 +/− mutant mice are kept on a CD1 outbred background. Dll4GOF mice are heterozigous double mutants for TetO7-Dll4 [28] and Tie2-rtTA-M2 [30], in C57/BL6 background. Transgene induction was performed 5 days before BM recovery, by adding doxycycline (4 mg/ml) (Sigma-Aldrich) in drinking water containing 4% sucrose (Sigma-Aldrich), ad libitum delivery.

### Mouse BM-VPC isolation

We obtained BM cells and isolated Lin- cells (which are negative for the following markers CD5, CD45R(B220), CD11b, Gr-1 (Ly-6G/C), 7-4, and Ter-119) using magnetic sorting (Miltenyi Biotec-MACS). For in vitro experiments, Lin- cells were isolated from balbC, Dll4+/−, Dll4GOF and respective WT counterparts. For in vivo experiments Lin- cells from Dll4+/− mice and corresponding WT counterparts were then sorted by FACS for Flk1 expression (PE conjugated Flk1 antibody from Pharmingen). Lin-Flk1+ cells (96–99% purity, as determined by FACS sorting) were defined as BM-VPC-WT or Dll4+/− BM-VPC and were injected intravenously (1×104 cells per injection) into mice without further culture.

To determine Dll4 induction by VEGF and SDF1, we cultured BM Lin- cells isolated from BalbC mice (with 4 to 6 weeks of age) in RPMI medium (Gibco) without any supplements in presence of VEGF (20 ng/ml, Sigma-Aldrich) and Heparin (5 U/ml, Sigma-Aldrich) or SDF1 (50 ng/ml, R and D Systems). To inhibit SDF1 activation we incubated BM-VPC with SDF1 and CXCR4 antagonist (5 µg/ml AMD3100, Sigma-Aldrich).

### In vitro co-culture assays

HUVEC were cultured at 1×104/cm2 cell density using EBM2 supplemented medium (Lonza) with 5% FBS (foetal bovine serum, Sigma-Aldrich), in 0,2% gelatine (Sigma-Aldrich) coated plates. After 24 h, medium was change to EBM2 supplemented medium (Lonza) with 2% FBS (foetal bovine serum, Sigma-Aldrich) and 1×105 mBM-VPC from Dll4+/−, Dll4GOF and respective WT, were put over cultured HUVEC monolayer. Cell contact was maintained for 18 h. After this period mBM-VPC were washed from the cultures and HUVECs collected for mRNA extraction. Neutralizing antibodies anti-mDll4 were added to co-cultures at 50 ug/ml (kindly provided by Dr. Hideo Yagita). The cultures using mDll4GOF BM-VPC and respective controls were maintained in presence of doxycyclin (1 µg/ml) (Sigma-Aldrich) for transgene induction.

### Endothelial cell biology microarray

We used Oligo GEArrays HybTube Format from SABiosciences. Total mRNA was isolated from HUVEC obtained from co-culture experiments with BM-VPC-Dll4+/−, MDll4GOF BM-VPC and respective WT counterparts. mRNA was obtained from 3 independent experiments and pooled together in similar proportions. cDNA synthetisis and array hybridization was performed as recommended by manufacturer instructions.

### Gene expression by RQ-PCR

mRNA levels were measured by real time RQ-PCR on the ABI Prism® 7900HT Sequence Detection System (Applied Biosystems) using the following primers and probes: hHey2 hHes1 Fibronectin1 VE-Cadherin hICAM2, mDll4. The housekeeping gene used to normalize the samples was 18S (human18S rRNA - 20x, Applied Biosystems) or ß-actin (agccatgtacgtagccatcc; ctctcagctgtggtggtgaa) (mouse). Each sample was analyzed in triplicate and each PCR experiment included at least one non-template control well. Membrane arrays were analysed using ImageJ.

### *In vivo* tumor formation assays

We performed two types of in vivo assays, one reconstituting mouse bone marrow with mBM-VPC with altered expression of Dll4 and the other introducing exogenous human progenitor cells expressing Dll4. In the first case, we used female NOD-SCID mice as recipients. NOD-SCID mice were sublethally irradiated (200 rads) and were intravenously injected after 24 hours with BM-VPC-WT or BM-VP C-dll4+/− (n = 6 in each experiment, for each condition). Control mice were not injected with BM-VPC. After three days, controls, BM-VPC-WT and BM-VPC-dll4+/− reconstituted mice were subcutaneously injected with 6×106 Human HL60 cell line (myeloid leukemia, which forms chloromas). Tumor volume was determined at different time points. The mice were sacrificed 15 days or 25 days after tumor implantation and blood samples were collected. Tumors were frozen and fixed for subsequent analysis. In the second in vivo assay, we used NOD-SCID mice that were subcutaneously injected with 4×106 mouse breast cancer cell line (HTH-K, [31], [32]). After 6 days, we divided the tumor injected mice into three groups, no cell treatment (n = 4), WT BM-VPC treated mice (n = 4) and mDll4GOF BM-VPC treated mice (n = 4). BM-VPC (3×105 cells per injection) were administered intravenously (obtained from mDll4GOF mice bone-marrow by Ficoll density gradient centrifugation and isolated as Lin- population; see BM-VPC isolation) at day 6, 8 and 10 after tumor inoculation. Mice were killed at day 11 after tumor inoculation.

### Fluorescent in situ hybridization (FISH)

Fixed blood samples and frozen BM sections were used. Frozen sections were rehydrated in PBS, then hybridized with a specific probe for mouse Y chromosome (Cambrio, UK). Frozen sections and blood samples were denaturation at 85°C for 5 minutes. Hybridization was carried out at 37°C overnight.

### Tumor microvessel determination, TUNEL, Proliferation, Hypoxia

Tumours cryosections were blocked with a 5% FBS/0,1% BSA solution in PBS for 30 minutes. Slides were then covered with primary antibodies (rat anti-PECAM from Pharmingen), mouse anti-alpha-SMA (DAKO) or rabbit anti-Histone 3 (Chemicon) overnight at 4°C. After 3 brief washes in PBS, secondary antibodies from Invitrogen (anti-rat-FITC, anti-mouse-Alexa568, anti-rabbit-Alexa488, respectively) and incubated for 2 hours. For the quantification of stable (CD31+ and SMA+) versus unstable (CD31+SMA-) vessels, stained sections were visualized, and the total number of vessels was determined in 5 high power fields (x400 magnification). For hypoxia determination we used Hypoxyprobe kit (Chemicon) and performed the immunostaining as indicated by the manufacturer. After briefly washing the slides in PBS, the slides were mounted in fluorescence mounting medium from DAKO. Slides were photographed using a standard fluorescence microscope. Hypoxia tumor area was determined by visualizing stained sections and quantifying the number of stained/unstained cells in 5 high power fields (x400 magnification).

### Statistical Analysis

Differences in tumor growth, proliferation, apoptosis, hypoxia and stable versus unstable vessels were analysed by ANOVA.

## References

[1] Asahara T, Masuda H, Takahashi T, Kalka C, Pastore C (1999). Bone marrow origin of endothelial progenitor cells responsible for postnatal vasculogenesis in physiological and pathological neovascularization.. Circ Res.

[2] Rafii S, Lyden D, Benezra R, Hattori K, Heissig B (2002). Vascular and haematopoietic stem cells: novel targets for anti-angiogenesis therapy?. Nat Rev Cancer.

[3] Lyden D, Hattori K, Dias S, Costa C, Blaikie P (2001). Impaired recruitment of bone-marrow-derived endothelial and hematopoietic precursor cells blocks tumor angiogenesis and growth.. Nat Med.

[4] Larrivee B, Niessen K, Pollet I, Corbel SY, Long M (2005). Minimal contribution of marrow-derived endothelial precursors to tumor vasculature.. J Immunol.

[5] Peters BA, Diaz LA, Polyak K, Meszler L, Romans K (2005). Contribution of bone marrow-derived endothelial cells to human tumor vasculature.. Nat Med.

[6] Pasquier E, Dias S (2010). Endothelial progenitor cells: hope beyond controversy.. Curr Cancer Drug Targets.

[7] Dudley AC, Udagawa T, Melero-Martin JM, Shih SC, Curatolo A (2010). Bone marrow is a reservoir for proangiogenic myelomonocytic cells but not endothelial cells in spontaneous tumors.. Blood.

[8] Krebs LT, Xue Y, Norton CR, Shutter JR, Maguire M (2000). Notch signaling is essential for vascular morphogenesis in mice.. Genes Dev.

[9] Uyttendaele H, Ho J, Rossant J, Kitajewski J (2001). Vascular patterning defects associated with expression of activated Notch4 in embryonic endothelium.. Proc Natl Acad Sci U S A.

[10] Duarte A, Hirashima M, Benedito R, Trindade A, Diniz P (2004). Dosage-sensitive requirement for mouse Dll4 in artery development.. Genes Dev.

[11] Gale NW, Dominguez MG, Noguera I, Pan L, Hughes V (2004). Haploinsufficiency of delta-like 4 ligand results in embryonic lethality due to major defects in arterial and vascular development.. Proc Natl Acad Sci U S A.

[12] Carmeliet P, Ferreira V, Breier G, Pollefeyt S, Kieckens L (1996). Abnormal blood vessel development and lethality in embryos lacking a single VEGF allele.. Nature.

[13] Mailhos C, Modlich U, Lewis J, Harris A, Bicknell R (2001). Delta4, an endothelial specific notch ligand expressed at sites of physiological and tumor angiogenesis.. Differentiation.

[14] Oliner J, Min H, Leal J, Yu D, Rao S (2004). Suppression of angiogenesis and tumor growth by selective inhibition of angiopoietin-2.. Cancer Cell.

[15] Patel NS, Li JL, Generali D, Poulsom R, Cranston DW (2005). Up-regulation of delta-like 4 ligand in human tumor vasculature and the role of basal expression in endothelial cell function.. Cancer Res.

[16] Williams CK, Li JL, Murga M, Harris AL, Tosato G (2006). Up-regulation of the Notch ligand Delta-like 4 inhibits VEGF-induced endothelial cell function.. Blood.

[17] Caiado F, Real C, Carvalho T, Dias S (2008). Notch pathway modulation on bone marrow-derived vascular precursor cells regulates their angiogenic and wound healing potential.. PLoS One.

[18] Escudier B, Eisen T, Stadler WM, Szczylik C, Oudard S (2007). Sorafenib in advanced clear-cell renal-cell carcinoma.. N Engl J Med.

[19] Llovet JM, Ricci S, Mazzaferro V, Hilgard P, Gane E (2008). Sorafenib in advanced hepatocellular carcinoma.. N Engl J Med.

[20] Motzer RJ, Bukowski RM (2006). Targeted therapy for metastatic renal cell carcinoma.. J Clin Oncol.

[21] Bergers G, Hanahan D (2008). Modes of resistance to anti-angiogenic therapy.. Nat Rev Cancer.

[22] Verheul HM, Pinedo HM (2007). Possible molecular mechanisms involved in the toxicity of angiogenesis inhibition.. Nat Rev Cancer.

[23] Noguera-Troise I, Daly C, Papadopoulos NJ, Coetzee S, Boland P (2006). Blockade of Dll4 inhibits tumour growth by promoting non-productive angiogenesis.. Nature.

[24] Ridgway J, Zhang G, Wu Y, Stawicki S, Liang WC (2006). Inhibition of Dll4 signalling inhibits tumour growth by deregulating angiogenesis.. Nature.

[25] Moore MA, Hattori K, Heissig B, Shieh JH, Dias S (2001). Mobilization of endothelial and hematopoietic stem and progenitor cells by adenovector-mediated elevation of serum levels of SDF-1, VEGF, and angiopoietin-1.. Ann N Y Acad Sci.

[26] Yamaguchi J, Kusano KF, Masuo O, Kawamoto A, Silver M (2003). Stromal cell-derived factor-1 effects on ex vivo expanded endothelial progenitor cell recruitment for ischemic neovascularization.. Circulation.

[27] Leslie JD, Ariza-McNaughton L, Bermange AL, McAdow R, Johnson SL (2007). Endothelial signalling by the Notch ligand Delta-like 4 restricts angiogenesis.. Development.

[28] Trindade A, Kumar SR, Scehnet JS, Lopes-da-Costa L, Becker J (2008). Overexpression of delta-like 4 induces arterialization and attenuates vessel formation in developing mouse embryos.. Blood.

[29] Huang MT, Mason JC, Birdsey GM, Amsellem V, Gerwin N (2005). Endothelial intercellular adhesion molecule (ICAM)-2 regulates angiogenesis.. Blood.

[30] Deutsch U, Schlaeger TM, Dehouck B, Doring A, Tauber S (2008). Inducible endothelial cell-specific gene expression in transgenic mouse embryos and adult mice.. Exp Cell Res.

[31] Dias S, Thomas H, Balkwill F (1998). Multiple molecular and cellular changes associated with tumour stasis and regression during IL-12 therapy of a murine breast cancer model.. Int J Cancer.

[32] Scott KA, Holdsworth H, Balkwill FR, Dias S (2000). Exploiting changes in the tumour microenvironment with sequential cytokine and matrix metalloprotease inhibitor treatment in a murine breast cancer model.. Br J Cancer.

